# Association of prepregnancy body mass index, rate of gestational weight gain with pregnancy outcomes in Chinese urban women

**DOI:** 10.1186/s12986-019-0386-z

**Published:** 2019-08-19

**Authors:** Xueyin Wang, Xiaosong Zhang, Min Zhou, Juan Juan, Xu Wang

**Affiliations:** Department of Obstetrics and Gynecology, Peking University First Hospital, No. 1 Xi’anmen Street, Xicheng District, Beijing, 100034 China

**Keywords:** Gestational weight gain, Body mass index, Pregnancy outcomes, Preterm birth, Cesarean section, Small-for-gestational age, Large-for-gestational age, Low birth weight, Macrosomia

## Abstract

**Background:**

The prevalence of obesity and excessive gestational weight gain (GWG) has been increasing worldwide. The aims of this study were to evaluate associations of prepregnancy body mass index (BMI) and rate of GWG in the 2nd and 3rd trimesters with pregnancy outcomes in Chinese urban women, and to examine the dose-response relationship between rate of GWG and pregnancy outcomes.

**Methods:**

A retrospective analysis included 8926 women who delivered live singletons at ≥28 weeks of gestation between June 2012 and March 2013 among Chinese urban women. BMI was classified into underweight (BMI < 18.5 kg/m^2^), normal weight (18.5 kg/m^2^ ≤ BMI < 24 kg/m^2^), overweight (24 kg/m^2^ ≤ BMI < 28 kg/m^2^) and obese (BMI ≥ 28 kg/m^2^) according to the Chinese standard. Rate of GWG in the 2nd and 3rd trimesters was classified as insufficient, adequate and excessive if it was below, within, or above the 2009 IOM guidelines (0.44–0.58 kg/w [underweight], 0.35–0.50 kg/w [normal], 0.23–0.33 kg/w [overweight], and 0.17–0.27 kg/w [obese]). Logistic regression models and restricted cubic spline analyses were used to assess the association of prepregnancy BMI and rate of GWG with cesarean delivery, preterm birth, small-for-gestational age (SGA) and large-for-gestational age (LGA).

**Results:**

22.6 and 50.0% of women had insufficient and excessive rate of GWG, respectively. After adjustment for potential confounders, prepregnancy underweight was associated with increased risk of SGA (OR = 1.71, 95% CI: 1.40–2.09), while both overweight and obesity were associated with higher risk of cesarean delivery (overweight: OR [95% CI] = 1.80 [1.56–2.08]; obese: 2.34 [1.69–3.24]) and LGA (overweight: 1.75 [1.44–2.13]; obese: 2.48 [1.71–3.60]). Both insufficient (OR = 1.34, 95% CI: 1.08–1.65) and excessive rates of GWG (OR = 1.44, 95% CI: 1.20–1.73) were associated with higher risk of preterm birth. Insufficient rate of GWG was associated with increased odds of SGA (OR = 1.49, 95% CI: 1.16–1.82), while excessive rate of GWG was associated with higher risk for cesarean delivery (OR = 1.22, 95% CI: 1.10–1.35) and LGA (OR = 1.58, 95% CI: 1.33–1.87). Additionally, there were significant nonlinear associations between rate of GWG and preterm birth (U-shaped, *P* for nonlinear < 0.001).

**Conclusions:**

Prepregnancy overweight, obesity and underweight, and insufficient and excessive rate of GWG were associated with increased risk of pregnancy outcomes in Chinese urban women.

**Electronic supplementary material:**

The online version of this article (10.1186/s12986-019-0386-z) contains supplementary material, which is available to authorized users.

## Background

Obesity and overweight have become major health concerns in women of childbearing age, and their prevalence has been increasing worldwide. Data from the Pregnancy Risk Assessment Monitoring System (PRAMS) indicated that 45% of US women who give birth were overweight and obese before pregnancy [1], while overweight and obesity have affected 6~24% of Chinese women of reproductive age [2–5]. Prepregnancy overweight or obesity has been associated with a number of poor maternal and neonatal outcomes such as higher risk of cesarean delivery, gestational diabetes mellitus (GDM), pregnancy-induced hypertension (PIH), preterm birth, macrosomia, and low Apgar scores [1, 4, 6]. In addition, maternal obesity may also affect the long-term health of their children, such as increasing the risk of obesity, poor body fat distribution, high blood pressure, adverse lipid profile, and insulin resistance in child and adult offspring [7, 8]. On the other hand, maternal underweight with the prevalence of 11–14% in Chinese pregnant women has been also associated with suboptimal fetal growth, including low birth weight and small-for-gestational age (SGA) [2–4, 6, 9].

Gestational weight gain (GWG) is a potentially modifiable risk factor for a series of adverse pregnancy outcomes which could be reduced by nutrition or exercise interventions during pregnancy [10]. Recently, a systematic review and meta-analysis estimated that 47% of pregnant women had excessive GWG and 23% had inadequate GWG according to 2009 Institute of Medicine (IOM) recommendations [11]. Excessive GWG has been associated with increased risk of cesarean delivery, hypertensive diseases of pregnancy, postpartum weight retention, macrosomia, and childhood overweight or obesity for the offspring, whereas insufficient GWG may contribute to low birth weight, preterm birth and failure to initiate breast-feeding [10, 12, 13]. In addition, some previous epidemiologic studies using total GWG (kg) as the measurement may introduce bias because of neglecting the inherent correlation between GWG and gestational age at delivery [14, 15]. Moreover, the rate of GWG is constant and its association with pregnancy time is close to be linear in the 2nd and 3rd trimesters [16], and thus, the rate of GWG in the 2nd and 3rd trimesters would be more preferable for evaluating the association between GWG and pregnancy outcomes. Furthermore, few studies have examined the dose-response relationship between GWG and pregnancy outcomes. To our knowledge, only two studies reported the dose-response relationship between rate of GWG in the 2nd and 3rd trimesters and preterm birth [15, 17].

Therefore, the purpose of the present study was to examine associations of prepregnancy body mass index (BMI) and rate of GWG in the 2nd and 3rd trimesters with pregnancy outcomes in Chinese urban women, and we also assessed the dose-response relationship between rate of GWG and pregnancy outcomes in the whole population and in women stratified by prepregnancy BMI categories.

## Methods

### Study design and participants

This was a multicenter, retrospective cohort study of postpartum women and their neonates conducted in 14 hospitals in urban areas of China, including 3 centers in Beijing, 2 centers in Guangdong province, 3 centers in Hunan province, 2 centers in Hubei province, 2 centers in Sichuan province and 2 centers in Shanxi province. Women aged ≥18 years with a gestational age of ≥28 weeks and live birth who delivered during 10th–19th of the last month of every quarter from June 2012 to March 2013 were recruited in order to control for seasonal variations. A structured questionnaire was designed to collect information on demographic characteristics, lifestyle behavior, obstetric and medical history of pregnancy, and pregnancy outcomes. During hospitalization for delivery, face-to-face interviews were conducted to collect information on demographic characteristics and lifestyle behavior, and clinical information was obtained retrospectively based on their medical records. Among 9152 participants with full medical history of regularly scheduled antenatal visits and delivery, 226 women with multiple gestation, pre-conception history of severe heart disease or chronic renal disease were excluded. Overall, the current analysis was limited to 8926 deliveries. All participants provided written informed consent, and the study was approved by the institutional review board of Peking University First Hospital.

### Classification of prepregnancy BMI and rate of GWG

The weight (kilograms) and height (meters) of all pregnant women were measured at each antenatal visits in light clothing without shoes. At the enrollment after delivery, weight and height at the first antenatal visit, weight at the last antenatal visit in the first trimester or the first antenatal visit in the 2nd trimester, and weight at the last antenatal visit or the time of delivery were recorded based on the medical records. Prepregnancy BMI was calculated as the weight in kilograms divided by the square of height measured in meters, and classified into four groups according to the Chinese standard [18]: underweight (BMI < 18.5 kg/m^2^), normal weight (18.5 kg/m^2^ ≤ BMI < 24 kg/m^2^), overweight (24 kg/m^2^ ≤ BMI < 28 kg/m^2^) and obese (BMI ≥ 28 kg/m^2^). The rate of GWG in the 2nd and 3rd trimesters was calculated as: [(final weight measured at the last antenatal visit or the time of delivery – weight measured at the last antenatal visit in the first trimester or the first antenatal visit in the 2nd trimester) / (gestational age at delivery – 13 weeks)] [16]. According to the 2009 IOM guidelines, the rate of GWG was classified into insufficient, adequate and excessive if it was below, within, or above the recommendations as follows: 0.44–0.58 kg/w (underweight), 0.35–0.50 kg/w (normal), 0.23–0.33 kg/w (overweight), and 0.17–0.27 kg/w (obese) [16].

### Definition of pregnancy outcomes

The main outcomes of this study were cesarean delivery, preterm birth (defined as delivery before 37 weeks gestation [19]), large-for-gestational age (LGA) and SGA. LGA and SGA were indicated by birth weight less than and greater than the 10th and 90th percentile, respectively, for the same gestational age by sex, according to the Chinese neonatal birth weight curve [20].

### Assessment of covariates

Covariates included age, gestational age at delivery, education, drinking during pregnancy, passive smoking, annual household income, number of parity, GDM, and PIH. Age, education, drinking during pregnancy, passive smoking, annual household income and number of parity were assessed by the interviewer-administered questionnaire. Gestational age at delivery was determined from the date of last menstrual period to the date of delivery and expressed in the week after the last menstrual period. If the date was uncertain, ultrasonography was used to determine gestational age. GDM was diagnosed according to the diagnostic criteria amended by China’s Ministry of Health [21], which recommend that the diagnosis should be made when any one of the following values is met or exceeded in the 75 oral glucose tolerance test at 24–28 weeks: 0 h, 5.1 mmol/L; 1 h, 10.0 mmol/L; and 2 h 8.5 mmol/L. PIH was defined as systolic blood pressure ≥ 140 mmHg or diastolic blood pressure ≥ 90 mmHg after 20 weeks of gestation [22].

### Statistical analysis

Demographics characteristics and pregnancy outcomes were presented as numbers and frequency distributions for categorical variables, or median and interquartile range for continuous variables, and were compared using the chi-square test or Kruskal-Wallis test. Multivariable logistic regression models were conducted to estimate odds ratios (ORs) and their 95% confidence intervals (CIs) of pregnancy outcomes across categories of prepregnancy BMI or rate of GWG. Models were adjusted for age, gestational age at delivery (except for the outcome of preterm birth), education, drinking during pregnancy, passive smoking, annual household income, number of parity, and study centers. Further models included mutual adjustment of prepregnancy BMI or rate of GWG as appropriate. The normal weight group and adequate GWG rate group were used as the reference groups, respectively. Sensitivity analyses were conducted to additionally adjust for GDM and PIH. Restricted cubic spline (RCS) logistic regression models were used to assess nonlinear effects of GWG rate on adverse pregnancy outcomes by treating the median level of GWG rate as the reference, using 4 knots located at the 5th, 35th, 65th, and 95th percentiles of the distribution of GWG rate. Analyses were carried out using SAS software version 9.2 (SAS Institute, Cary, NC), and the RCS was performed using SAS *RCS_Reg* macro [23]. All *P* values are two-sided, and a 0.05 level was used to declare significant differences.

## Results

### Participant characteristics

**Fig. 1:**
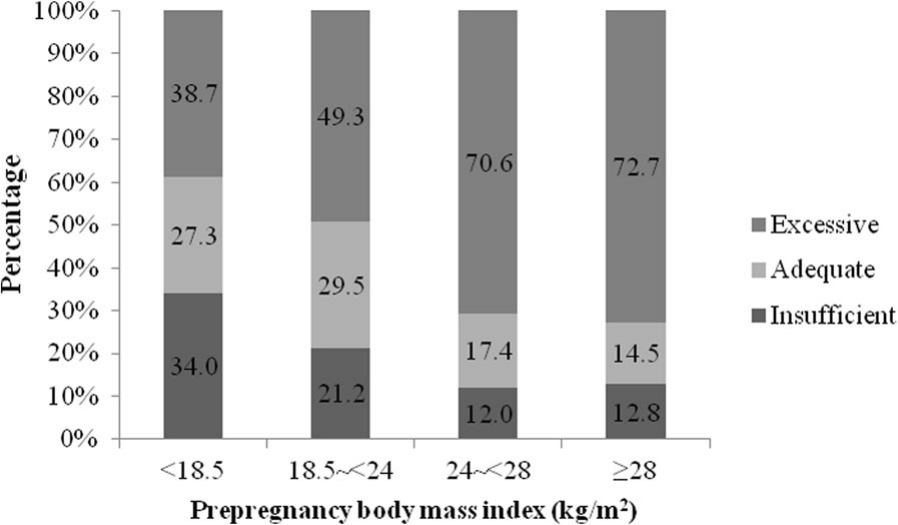
Percentage of gestational weight gain rate categories in different prepregnancy BMI groups

Among 8926 postpartum women, 22.6, 27.4 and 50.0% of women had insufficient, adequate and excessive rate of GWG, respectively. More than 70% of participants who were overweight or obese had excessive rate of GWG (Fig. 1). Distribution of pregnancy outcomes, prepregnancy BMI and rate of GWG by study centers is shown in Additional file 1: Table S1 and Additional file 2: Table S2, respectively. Table 1 shows demographic and clinical characteristics of study participants according to the prepregnancy BMI and rate of GWG categories. The prepregnancy BMI was inversely associated with rate of GWG (*P* < 0.001). Women with greater prepregnancy BMI were more likely to be multiparous, and have a higher risk of GDM and PIH, and were less likely to have a higher level of annual household income (all *P* < 0.05). Overweight women were slightly older and obese women were more likely to be exposed to passive smoking than women of normal weight (all *P* < 0.05). Women with excessive rate of GWG had a higher likelihood of PIH, and women with insufficient rate of GWG were more likely to be multiparous, and to have a lower education level than those with adequate rate of GWG (all *P* < 0.05).

**Table 1:** Characteristics of participants by prepregnancy BMI and rate of gestational weight gain

	Prepregnancy BMI					Gestational weight gain rate			
	Underweight	Normal weight	Overweight	Obese	*P* value	Insufficient	Adequate	Excessive	*P* value
Participants, n (%)	1787 (20.0)	6006 (67.3)	961 (10.8)	172 (1.9)		2018 (22.6)	2449 (27.4)	4459 (50.0)	
prepregnancy BMI, kg/m^2^, median (IQR)	17.6 (16.9,18.1)	20.6 (19.5,21.9)	25.1 (24.4,26.0)	29.4 (28.7,31.2)	< 0.001	20.0 (18.3, 22.0)	20.2 (18.8,22.0)	20.5 (19.1,22.8)	< 0.001
Gestational weight gain rate, kg/w, median (IQR)	0.42 (0.34,0.51)	0.40 (0.33,0.50)	0.35 (0.26,0.44)	0.29 (0.21,0.41)	< 0.001	0.27 (0.19, 0.33)	0.44 (0.38,0.48)	0.63 (0.56,0.75)	< 0.001
Age, years, median (IQR)	27 (25,30)	28 (26,31)	29 (27,32)	28 (26,31)	< 0.001	28 (25, 31)	28 (26, 31)	28 (26, 30)	0.005
Gestational age at delivery, week	39 (38, 40)	39 (38,40)	39 (38,40)	39 (38,40)	0.183	39 (38, 40)	39 (38, 40)	39 (38, 40)	< 0.001
Education					0.205				< 0.001
High school and below	1198 (67.0)	3914 (65.2)	650 (67.6)	119 (69.2)		1415 (70.1)	1576 (64.4)	2890 (64.8)	
College or graduate school	589 (33.0)	2092 (34.8)	311 (32.4)	53 (30.8)		603 (29.9)	873 (35.6)	1569 (35.2)	
Drinking during pregnancy					0.570				0.084
Yes	52 (2.9)	160 (2.7)	24 (2.5)	2 (1.2)		40 (2.0)	67 (2.7)	131 (2.9)	
No	1735 (97.1)	5846 (97.3)	937 (97.5)	170 (98.8)		1978 (98.0)	2382 (97.3)	4328 (97.1)	
Smoking during pregnancy					0.358				0.904
Yes	5 (0.3)	37 (0.6)	4 (0.4)	1 (0.6)		11 (0.6)	14 (0.6)	22 (0.5)	
No	1782 (99.7)	5969 (99.4)	957 (99.6)	171 (99.4)		2007 (99.4)	2435 (99.4)	4437 (99.5)	
Passive smoking					< 0.001				0.721
Yes	408 (22.8)	1182 (19.7)	167 (17.4)	44 (25.6)		420 (20.8)	489 (20.0)	892 (20.0)	
No	1379 (77.2)	4824 (80.3)	794 (82.6)	128 (74.4)		1598 (79.2)	1960 (80.0)	3567 (80.0)	
Annual household income, yuan					0.009				0.752
< 10,000	1426 (79.8)	4799 (79.9)	766 (79.7)	147 (85.5)		1609 (79.7)	1961 (80.1)	3568 (80.0)	
10,001–20,000	239 (13.4)	885 (14.7)	156 (16.2)	20 (11.6)		306 (15.2)	358 (14.6)	636 (14.3)	
> 20,000	122 (6.8)	322 (5.4)	39 (4.1)	5 (2.9)		103 (5.1)	130 (5.3)	255 (5.7)	
Number of parity					< 0.001				< 0.001
Primiparous	1552 (86.9)	4904 (81.7)	710 (73.9)	125 (72.7)		1559 (77.3)	2001 (81.7)	3731 (83.7)	
Multiparous	235 (13.1)	1102 (18.3)	251 (26.1)	47 (27.3)		459 (22.7)	448 (18.3)	728 (16.3)	
Gestational diabetes mellitus					< 0.001				0.279
Yes	100 (5.6)	539 (9.0)	163 (17.0)	52 (30.2)		176 (8.7)	233 (9.5)	445 (10.0)	
No	1687 (94.4)	5467 (91.0)	798 (83.0)	120 (69.8)		1842 (91.3)	2216 (90.5)	4014 (90.0)	
PIH					< 0.001				< 0.001
Yes	44 (2.5)	237 (3.9)	82 (8.5)	29 (16.9)		52 (2.6)	84 (3.4)	256 (5.7)	
No	1743 (97.5)	5769 (96.1)	879 (91.5)	143 (83.1)		1966 (97.4)	2365 (96.6)	4203 (94.3)	

Abbreviations: *BMI* body mass index, *GWG* gestational weight gain

Values are median (IOR) or n (%)

Additional file 3: Table S3 illustrates the prevalence of adverse pregnancy outcomes by prepregnancy BMI and rate of GWG categories. Prepregnancy BMI and rate of GWG were positively associated with risk of cesarean delivery and LGA (all *P* < 0.001). Women with underweight and obese had higher prevalence of SGA compared with those of normal weight (*P* < 0.001). Both insufficient and excessive rates of GWG were associated with higher prevalence of preterm birth than those with adequate rate of GWG (all *P* < 0.01).

### Associations of prepregnancy BMI and rate of GWG with pregnancy outcomes

Table 2 shows associations of adverse pregnancy outcomes with prepregnancy BMI and rate of GWG categories. After adjustment for potential confounders, prepregnancy underweight was associated with higher risk for SGA (OR = 1.71, 95% CI: 1.40–2.09), and both prepregnancy overweight and obesity were associated with higher risk for cesarean delivery (overweight: OR = 1.80, 95% CI: 1.56–2.08; obese: OR = 2.34, 95% CI: 1.69–3.24), and LGA (overweight: OR = 1.75, 95% CI: 1.44–2.13; obese: OR = 2.48, 95% CI: 1.71–3.60). Insufficient rate of GWG was associated with increased odds of preterm birth (OR = 1.34, 95% CI: 1.08–1.65), and SGA (OR = 1.45, 95% CI: 1.16–1.82), while excessive rate of GWG was associated with higher risk for cesarean delivery (OR = 1.22, 95% CI: 1.10–1.35), preterm birth (OR = 1.44, 95% CI: 1.20–1.73), and LGA (OR = 1.58, 95% CI: 1.33–1.87). The results remained significant after further mutual adjustment for rate of GWG or prepregnancy BMI.

**Table 2:** Adjusted ORs (95% CIs) for pregnancy outcomes by prepregnancy BMI and rate of GWG

Outcome	Prepregnancy BMI^a^				Rate of gestational weight gain^b^		
	Underweight	Normal weight	Overweight	Obese	Insufficient	Adequate	Excessive
Cesarean delivery							
Case/Non-case	765/1022	2810/3196	582/379	115/58	890/1128	1131/1318	2250/2209
Model 1	0.87 (0.78,0.97)	Reference	1.80 (1.56,2.08)	2.34 (1.69,3.24)	0.91 (0.81,1.03)	Reference	1.22 (1.10,1.35)
Model 2	0.86 (0.77,0.97)	Reference	1.87 (1.62,2.16)	2.50 (1.80,3.47)	0.93 (0.82,1.05)	Reference	1.15 (1.04,1.28)
Preterm birth^c^							
Case/Non-case	176/1611	528/5478	92/869	17/155	203/1815	180/2269	430/4029
Model 1	1.16 (0.97,1.40)	Reference	1.12 (0.88,1.42)	1.18 (0.70,1.98)	1.34 (1.08,1.65)	Reference	1.44 (1.20,1.73)
Model 2	1.15 (0.96,1.39)	Reference	1.19 (0.94,1.52)	1.32 (0.79,2.23)	1.34 (1.08,1.66)	Reference	1.43 (1.19,1.72)
SGA							
Case/Non-case	161/1626	331/5675	50/911	12/160	187/1831	158/2291	209/4250
Model 1	1.71 (1.40,2.09)	Reference	0.97 (0.71,1.32)	1.42 (0.77,2.59)	1.45 (1.16,1.82)	Reference	0.75 (0.61,0.93)
Model 2	1.74 (1.42,2.13)	Reference	0.89 (0.65,1.21)	1.19 (0.65,2.19)	1.43 (1.15,1.79)	Reference	0.77 (0.62,0.96)
LGA							
Case/Non-case	117/1670	608/5398	156/805	40/132	133/1885	205/2244	583/3876
Model 1	0.60 (0.49,0.74)	Reference	1.75 (1.44,2.13)	2.48 (1.71,3.60)	0.78 (0.62,0.97)	Reference	1.58 (1.33,1.87)
Model 2	0.59 (0.48,0.72)	Reference	1.91 (1.57,2.33)	2.90 (1.99,4.23)	0.79 (0.62,0.99)	Reference	1.48 (1.24,1.75)

Abbreviations: *BMI* body mass index, *CI* confidence interval, *GWG* gestational weight gain, *OR* odds ratio, *SGA* small-for-gestational age, *LGA* large-for-gestational age

Values are odds ratios (95% confidence intervals). Model 1 was adjusted for study centers, age, gestational age at delivery, education, drinking during pregnancy, passive smoking, annual household income, number of parity; Model 2 was additionally adjusted for gestational weight gain (continuous)^a^ or prepregnancy BMI (continuous) ^b^; Preterm birth was not adjusted for gestational age at delivery^c^

Sensitivity analyses were conducted to additionally adjust for GDM and PIH, and results were generally similar between models including and not including these two variables (Additional file 4: Table S4). We further examined the relationships between rate of GWG and pregnancy outcomes by prepregnancy BMI categories. Results were generally consistent pertaining to associations of GWG rate with the risk of pregnancy outcomes across different BMI groups, except for cesarean delivery (Additional file 6: Figure S1).

### Dose-response relationship of rate of GWG and adverse pregnancy outcomes

**Fig. 2:**
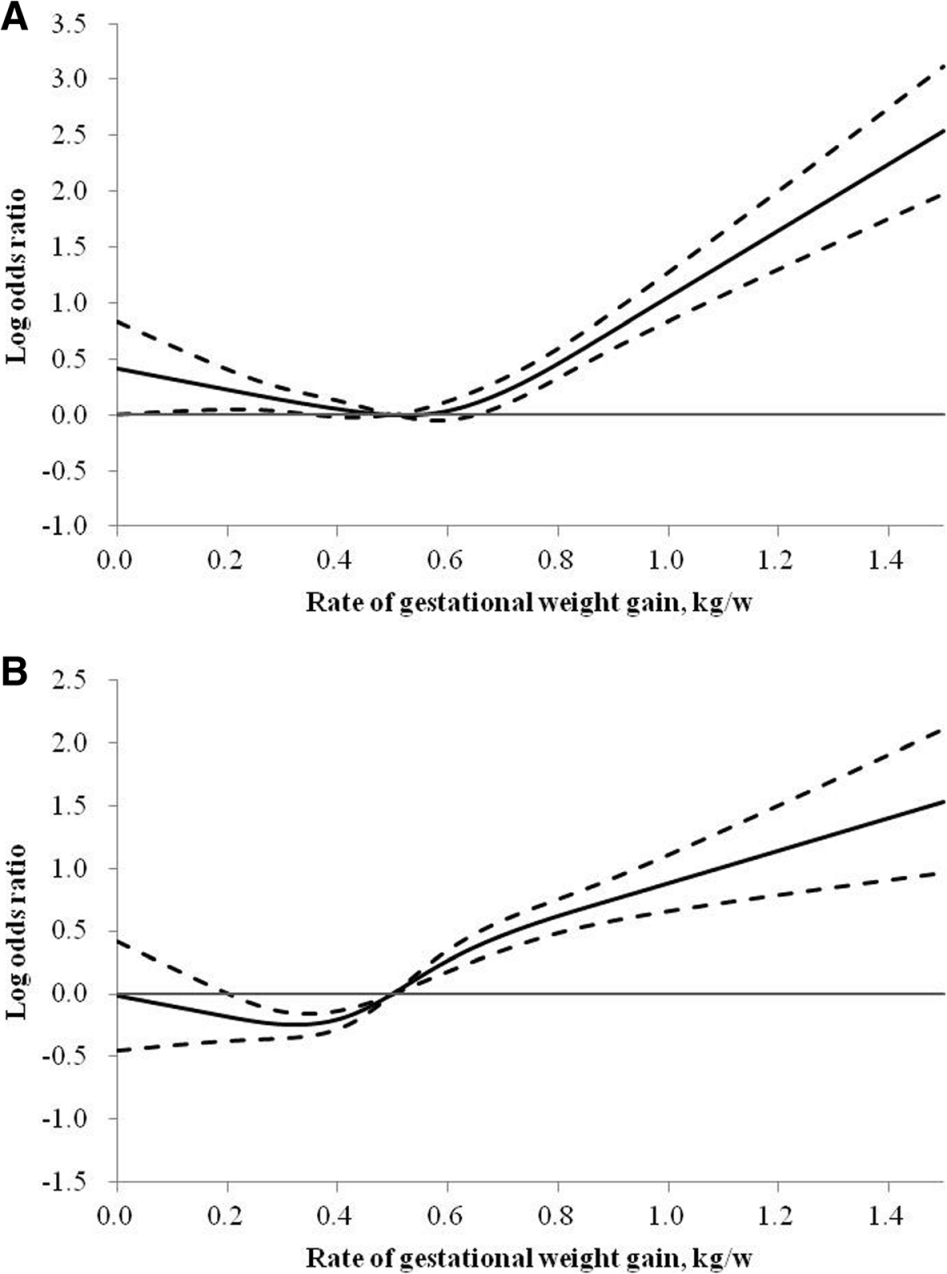
Log ORs and 95% CIs for rate of GWG with risk of preterm birth (**a**) and LGA (**b**). Abbreviations: BMI, body mass index; CI, confidence interval; GWG, gestational weight gain; LGA, large-for-gestational age; OR, odds ratio. Adjusted for study centers, age, gestational age at delivery, education, drinking during pregnancy, passive smoking, annual household income, number of parity, and prepregnancy BMI; Preterm birth was not adjusted for gestational age at delivery

The RCS analysis presented in Additional file 5: Table S5 partly confirmed the results above. Higher levels of GWG rate were associated with higher risk of cesarean delivery and LGA, and associated with lower risk of SGA (all *P*_overall_ < 0.001). Both lower and higher levels of GWG rate were associated with increased risk of preterm birth compared with the median of GWG rate of 0.50 kg/w (*P*_overall_ < 0.001, Additional file 5: Table S5 and Fig. 2). Fig. 2 shows the shape of associations between continuous rate of GWG and the risk of preterm birth and LGA based on the RCS logistic regression models. Among all participants, rate of GWG was nonlinearly associated with the risk of preterm birth (U-shaped, *P*_nonlinear_ < 0.001) and LGA (*P*_nonlinear_ = 0.002) after adjustment for potential confounders. Subgroup analysis based on different BMI categories has been shown in Additional file 5: Table S5. Similar results for the shape and the magnitude of associations of GWG rate with the risk of pregnancy outcomes were found between women with normal weight and the whole population.

## Discussion

In this retrospective cohort study of Chinese urban women, 22.6, 27.4 and 50.0% of women had insufficient, adequate and excessive rate of GWG in the 2nd and 3rd trimesters, respectively, and more than 70% of women who were overweight or obese had excessive rate of GWG according to the IOM recommendations. We also found that maternal underweight prepregnancy BMI was associated with higher risk of SGA, while overweight or obese pregnancy BMI was associated with increased odds of cesarean delivery and LGA. After adjusting for potential confounding factors, both insufficient and excessive rates of GWG were associated with higher risk of preterm birth than women with adequate rate of GWG. In addition, insufficient rate of GWG was associated with increased risk of SGA, whereas excessive rate of GWG was associated with higher risk of cesarean delivery and LGA.

One interesting finding of our study is that we observed a U-shaped association of GWG rate with preterm birth by both logistic regression and RCS analysis. Consistent with our findings, a previous study also reported a U-shaped relationship, with both low and high rates of 2nd and 3rd trimester GWG having a positive association with preterm birth among US pregnancy women [17]. Another study also indicated that the association between rate of GWG and preterm birth was U-shaped in Peruvian women who were normal-weight or overweight before pregnancy [24]. The potential mechanisms linking insufficient rate of GWG and an elevated risk of preterm birth may involve diminished uteroplacental blood flow, poor expansion of plasma volume, deficiencies in micronutrients, impaired antioxidant activity, infection and inflammation, while excessive rate of GWG may be associated with an increased risk of preterm birth due to induction of a pro-inflammatory state and fluid retention associated with preeclampsia [24, 25]. Potential mechanisms underlying the association between GWG and preterm birth needed to be further explored in future research.

Another finding of our study is that insufficient rate of GWG was associated with higher risk of SGA. In concordance with our results, the forementioned PRAMS cohort also reported that inadequate GWG in underweight and normal prepregnancy BMI groups was associated with increased risk for SGA infants [26]. A large-scale retrospective study conducted in Japanese women found that poor GWG was associated with a higher frequency of SGA [6]. Another retrospective cohort study focusing on Taipei women also demonstrated that GWG below the guideline increased the risk of SGA in women with prepregnancy underweight or normal weight [4]. In contrast to a previous study suggesting that GWG below recommendations was associated with a four-fold risk of SGA in US obese women [27], our study demonstrated that insufficient rate of GWG was associated with SGA among women who were prepregnancy underweight or normal weight rather than overweight/obese women. Differences in findings may be partly due to ethnicity disparities. Although factors associated with low GWG and high risk of SGA are largely unknown, several studies inferred that ethnicity, inflammation and preeclampsia might be risk factors for poor fetal growth [28].

The positive associations of prepregnancy BMI and rate of GWG with increased risk of LGA have reported in different populations [4, 6, 11, 26], which is in line with our study. A retrospective cohort study conducted in Taiwanese women also reported that overweight or obese women were at risk for LGA, compared with the women of normal weight, and GWG above the guideline was associated with higher rates of LGA [4]. Another study also revealed that excess GWG was associated with a higher frequency of LGA in Japanese women [6]. Similar to that reported by a recent systematic review and meta-analysis [11], we also found the strongest association between insufficient rate of GWG and lower risk of LGA in underweight women. It might be partly explained by the association of baseline maternal BMI and GWG with changes in the hormonal milieu, including insulin resistance, suggested by animal studies [29].

In addition, we also found that prepregnancy overweight/obesity was associated with an elevated risk of cesarean delivery, which was in accordance with the findings of previous studies [6, 30–32]. The association between overweight/obese and increased risk of cesarean delivery might partly due to excess pelvic soft tissue which can lead to a relative obstruction of the birth canal, and decreased rates of cervical dilation and subsequent increased rate of inductions after labor had started [31, 33]. However, the relationship between rate of GWG and cesarean delivery is still controversial. A prospective cohort study conducted in the Netherlands reported that 1st trimester rate of GWG increased odds of cesarean delivery (OR = 1.19, 95% CI 1.10–1.29), while rate of 2nd and 3rd trimester GWG was not associated with cesarean delivery [30]. Another retrospective cohort study among Hispanic women indicated that rate of GWG in the 3rd trimester was associated with a 1.24 odds of cesarean delivery, whereas excessive rate of GWG in the 1st and 2nd trimester was not associated with higher risk of cesarean delivery [31]. In the current study, we observed that rate of GWG in the 2nd and 3rd trimesters was associated with increased risk of cesarean delivery. Prior studies indicated that excessive GWG might contribute to risk of cesarean delivery through an increase in child birth weight, and an elevated rate of preeclampsia independent of prepregnancy BMI [31, 33].

The main strengths of this study include a relatively large sample size, using rate of GWG in the 2nd and 3rd trimesters to control for the effect of length of pregnancy on pregnancy outcomes, adjusting for potential confounding factors comprehensively, and utilizing RCS analysis to assess dose-response relationships of GWG rate with adverse pregnancy outcomes. Our study has several limitations. First, our data included only Chinese Han population, and it is unclear whether the results can be extrapolated to women of other ethnic groups. Second, we did not record GWG for different trimesters of pregnancy, so we were unable to examine associations of pregnancy outcomes with rate of GWG in different trimesters. Finally, we did not differentiate spontaneous and induced preterm birth, and did not distinguish between emergency and elective cesarean deliveries.

## Conclusion

In summary, our study indicated that overweight or obese women had higher risk of cesarean delivery and LGA, and underweight women had higher risk of SGA. We observed a U-shaped association between GWG rate in the 2nd and 3rd trimesters and preterm birth. And we found that insufficient rate of GWG was associated with increased risk of SGA, whereas excessive rate of GWG was associated with higher risk of cesarean delivery and LGA. Our findings emphasize the importance of maintaining normal prepregnancy BMI and adequate GWG in preventing adverse pregnancy outcomes by implementing healthy lifestyle strategies before and during pregnancy.

## Additional files

Additional file 1: **Table S1.** Distribution of pregnancy outcomes by study centers. (DOCX 31 kb)

Additional file 2: **Table S2.** Distribution of prepregnancy BMI and rate of gestational weight gain by study centers. (DOCX 20 kb)

Additional file 3: **Table S3.** Pregnancy outcomes by prepregnancy BMI and rate of gestational weight gain. (DOCX 17 kb)

Additional file 4: **Table S4.** Adjusted ORs (95% CIs) for pregnancy outcomes by prepregnancy BMI and rate of gestational weight gain. (DOCX 16 kb)

Additional file 5: **Table S5.** Adjusted ORs (95% CIs) for pregnancy outcomes and rate of gestational weight gain. (DOCX 19 kb)

Additional file 6: **Figure S1.** Adjusted ORs for pregnancy outcomes according to prepregnancy BMI and rate of gestational weight gain. Abbreviations: BMI: body mass index; GWG, gestational weight gain; LGA, large-for-gestational age; OR: odds ratio; SGA, small-for-gestational age. Values are ORs for cesarean delivery (A), preterm birth (B), SGA (C) and LGA (D). Adjusted for study centers, age, gestational age at delivery, education, drinking during pregnancy, passive smoking, annual household income, and number of parity; Preterm birth was not adjusted for gestational age at delivery^*^. (JPG 52 kb)

## Data Availability

The datasets analyzed during the current study are available from the corresponding author on reasonable request.

## Abbreviations

BMI: Body mass index
CI: Confidence interval
GDM: Gestational diabetes mellitus
GWG: Gestational weight gain
IOM: Institute of Medicine
LGA: Large-for-gestational age
OR: Odds ratio
PIH: Pregnancy-induced hypertension
PRAMS: Pregnancy Risk Assessment Monitoring System
RCS: Restricted cubic spline
SGA: Small-for-gestational age
