# Supplementary material for: Association of prepregnancy body mass index, rate of gestational weight gain with pregnancy outcomes in Chinese urban women
Source: Nutr Metab (Lond). 2019 Aug 19;16:54. doi: 10.1186/s12986-019-0386-z (PMC6700840; doi:10.1186/s12986-019-0386-z)
Supplement: Supplementary file 1 — Table S1. Distribution of pregnancy outcomes by study centers. (DOCX 31 kb) [file 12986_2019_386_MOESM1_ESM.docx]

**Table S1.** Distribution of pregnancy outcomes by study centers

| Study centers | n | Cesarean delivery | Preterm birth | SGA | LGA |
| --- | --- | --- | --- | --- | --- |
| 1 | 281 | 146 (52.0) | 22 (7.8) | 19 (6.8) | 30 (10.7) |
| 2 | 651 | 300 (46.1) | 39 (6.0) | 22 (3.4) | 74 (11.4) |
| 3 | 821 | 379 (46.2) | 45 (5.5) | 43 (5.2) | 134 (16.3) |
| 4 | 494 | 239 (48.4) | 39 (7.9) | 47 (9.5) | 33 (6.7) |
| 5 | 841 | 313 (37.2) | 81 (9.6) | 55 (6.5) | 54 (6.4) |
| 6 | 673 | 328 (48.7) | 57 (8.5) | 42 (6.2) | 67 (10.0) |
| 7 | 200 | 131 (65.5) | 41 (20.5) | 16 (8.0) | 23 (11.5) |
| 8 | 802 | 276 (34.4) | 45 (5.6) | 47 (5.9) | 87 (10.9) |
| 9 | 1099 | 536 (48.8) | 146 (13.3) | 54 (4.9) | 137 (12.5) |
| 10 | 1030 | 687 (66.7) | 112 (10.9) | 77 (7.5) | 93 (9.0) |
| 11 | 302 | 156 (51.7) | 22 (7.3) | 17 (5.6) | 21 (7.0) |
| 12 | 434 | 205 (47.2) | 43 (9.9) | 38 (8.8) | 48 (11.1) |
| 13 | 909 | 395 (43.5) | 63 (6.9) | 48 (5.3) | 87 (9.6) |
| 14 | 389 | 180 (46.3) | 58 (14.9) | 29 (7.5) | 33 (8.5) |
| *P* value |  | <0.001 | <0.001 | 0.001 | <0.001 |

Abbreviations: SGA, small-for-gestational age; LGA, large-for-gestational age.

List of 14 study centers: 1=Peking University First Hospital; 2=Shunyi Women’s & Children’s Hospital of Beijing Children’s Hospital; 3=Tongzhou Maternal & Child Health Hospital of Beijing; 4= Maternal and Child Hospital of Guangdong Province; 5= Shenzhen Maternity & Child Healthcare Hospital; 6= Hunan Province Maternal and Child Health Care Hospital; 7=Xiangya Hospital Central South University; 8=Changsha Hospital for Maternal & Child Health Care; 9=Maternal and Child Hospital of Hubei Province; 10=Wuhan Women and Children Care Center; 11=Sichuan Provincial Hospital for Women and Children; 12=Zigong Hospital for Maternal & Child Health Care; 13=Shanxi Provincial Hospital for Women and Children; 14=Baoji Hospital for Maternal & Child Health Care.
