# Supplementary material for: Association of prepregnancy body mass index, rate of gestational weight gain with pregnancy outcomes in Chinese urban women
Source: Nutr Metab (Lond). 2019 Aug 19;16:54. doi: 10.1186/s12986-019-0386-z (PMC6700840; doi:10.1186/s12986-019-0386-z)
Supplement: Supplementary file 2 — Table S2. Distribution of prepregnancy BMI and rate of gestational weight gain by study centers. (DOCX 20 kb) [file 12986_2019_386_MOESM2_ESM.docx]

**Table S2.** Distribution of prepregnancy BMI and rate of gestational weight gain by study centers

| Study centers | Prepregnancy BMI | | | |  | Rate of gestational weight gain | | |
| --- | --- | --- | --- | --- | --- | --- | --- | --- |
|  | Underweight | Normal weight | Overweight | Obese |  | Insufficient | Adequate | Excessive |
| 1 | 38 (13.5) | 191 (68.0) | 37 (13.2) | 15 (5.3) |  | 57 (20.3) | 89 (31.7) | 135 (48.0) |
| 2 | 115 (17.7) | 403 (61.9) | 101 (15.5) | 32 (4.9) |  | 86 (13.2) | 126 (19.4) | 439 (67.4) |
| 3 | 151 (18.4) | 521 (63.5) | 113 (13.8) | 36 (4.3) |  | 118 (14.4) | 189 (23.0) | 514 (62.6) |
| 4 | 119 (24.1) | 319 (64.6) | 49 (9.9) | 7 (1.4) |  | 167 (33.8) | 153 (31.0) | 174 (35.2) |
| 5 | 55 (6.5) | 601 (71.5) | 174 (20.7) | 11(1.3) |  | 234 (27.8) | 254 (30.2) | 353 (42.0) |
| 6 | 148 (22.0) | 456 (67.8) | 61 (9.1) | 8 (1.2) |  | 113 (16.8) | 170 (25.2) | 390 (58.0) |
| 7 | 36 (18.0) | 144 (72.0) | 18 (9.0) | 2 (1.0) |  | 48 (24.0) | 69 (34.5) | 83 (41.5) |
| 8 | 220 (27.4) | 521 (65.0) | 55 (6.9) | 6 (0.7) |  | 201 (25.0) | 194 (24.2) | 407 (50.8) |
| 9 | 272 (24.8) | 740 (67.3) | 71 (6.5) | 16 (1.4) |  | 251 (22.8) | 309 (28.1) | 539 (49.1) |
| 10 | 205 (19.9) | 720 (69.9) | 96 (9.3) | 9 (0.9) |  | 260 (25.2) | 282 (27.4) | 488 (47.4) |
| 11 | 70 (23.2) | 206 (68.2) | 23 (7.6) | 3 (1.0) |  | 87 (28.8) | 101 (33.4) | 114 (37.8) |
| 12 | 117 (27.0) | 286 (65.9) | 28 (6.4) | 3 (0.7) |  | 103 (23.7) | 130 (30.0) | 201 (46.3) |
| 13 | 166 (18.3) | 622 (68.4) | 104 (11.4) | 17 (1.9) |  | 195 (21.4) | 287 (31.6) | 427 (47.0) |
| 14 | 75 (19.3) | 276 (71.0) | 31 (7.9) | 7 (1.8) |  | 98 (25.2) | 96 (24.7) | 195 (50.1) |
| *P* value |  | <0.001 |  |  |  |  | <0.001 |  |

Abbreviations: BMI, body mass index; SGA, small-for-gestational age; LGA, large-for-gestational age.

List of 14 study centers: 1=Peking University First Hospital; 2=Shunyi Women’s & Children’s Hospital of Beijing Children’s Hospital; 3=Tongzhou Maternal & Child Health Hospital of Beijing; 4= Maternal and Child Hospital of Guangdong Province; 5= Shenzhen Maternity & Child Healthcare Hospital; 6= Hunan Province Maternal and Child Health Care Hospital; 7=Xiangya Hospital Central South University; 8=Changsha Hospital for Maternal & Child Health Care; 9=Maternal and Child Hospital of Hubei Province; 10=Wuhan Women and Children Care Center; 11=Sichuan Provincial Hospital for Women and Children; 12=Zigong Hospital for Maternal & Child Health Care; 13=Shanxi Provincial Hospital for Women and Children; 14=Baoji Hospital for Maternal & Child Health Care.
