# Supplementary material for: Association of prepregnancy body mass index, rate of gestational weight gain with pregnancy outcomes in Chinese urban women
Source: Nutr Metab (Lond). 2019 Aug 19;16:54. doi: 10.1186/s12986-019-0386-z (PMC6700840; doi:10.1186/s12986-019-0386-z)
Supplement: Supplementary file 3 — Table S3. Pregnancy outcomes by prepregnancy BMI and rate of gestational weight gain. (DOCX 17 kb) [file 12986_2019_386_MOESM3_ESM.docx]

**Table S3.** Pregnancy outcomes by prepregnancy BMI and rate of gestational weight gain

|  | Prepregnancy BMI | | | | |  | Rate of gestational weight gain | | | |
| --- | --- | --- | --- | --- | --- | --- | --- | --- | --- | --- |
|  | Underweight | Normal weight | Overweight | Obese | *P* value |  | Insufficient | Adequate | Excessive | *P* value |
| Cesarean delivery |  |  |  |  | <0.001 |  |  |  |  | <0.001 |
| Yes | 765 (42.8) | 2810 (46.8) | 582 (60.6) | 114 (66.3) |  |  | 890 (44.1) | 1131 (46.2) | 2250 (50.5) |  |
| No | 1022 (57.2) | 3196 (53.2) | 379 (39.4) | 58 (33.7) |  |  | 1128 (55.9) | 1318 (53.8) | 2209 (49.5) |  |
| Preterm birth |  |  |  |  | 0.515 |  |  |  |  | 0.002 |
| Yes | 176 (9.8) | 528 (8.8) | 92 (9.6) | 17 (9.9) |  |  | 203 (10.1) | 180 (7.3) | 430 (9.6) |  |
| No | 1611 (90.2) | 5478 (91.2) | 869 (90.4) | 155 (90.1) |  |  | 1815 (89.9) | 2269 (92.7) | 4029 (90.4) |  |
| SGA |  |  |  |  | <0.001 |  |  |  |  | <0.001 |
| Yes | 161 (9.0) | 331 (5.5) | 50 (5.2) | 12 (7.0) |  |  | 187 (9.3) | 158 (6.5) | 209 (4.7) |  |
| No | 1626 (91.0) | 5675 (94.5) | 911 (94.8) | 160 (93.0) |  |  | 1831 (90.7) | 2291 (93.6) | 4250 (95.3) |  |
| LGA |  |  |  |  | <0.001 |  |  |  |  | <0.001 |
| Yes | 117 (6.5) | 608 (10.1) | 156 (16.2) | 40 (23.3) |  |  | 133 (6.6) | 205 (8.4) | 583 (13.1) |  |
| No | 1670 (93.5) | 5398 (89.9) | 805 (83.8) | 132 (76.7) |  |  | 1885 (93.4) | 2244 (91.6) | 3876 (86.9) |  |

Abbreviations: BMI, body mass index; SGA, small-for-gestational age; LGA, large-for-gestational age.

Values are n (%).
