# Supplementary material for: Association of prepregnancy body mass index, rate of gestational weight gain with pregnancy outcomes in Chinese urban women
Source: Nutr Metab (Lond). 2019 Aug 19;16:54. doi: 10.1186/s12986-019-0386-z (PMC6700840; doi:10.1186/s12986-019-0386-z)
Supplement: Supplementary file 4 — Table S4. Adjusted ORs (95% CIs) for pregnancy outcomes by prepregnancy BMI and rate of gestational weight gain. (DOCX 16 kb) [file 12986_2019_386_MOESM4_ESM.docx]

**Table S4.** Adjusted ORs (95% CIs) for pregnancy outcomes by prepregnancy BMI and rate of gestational weight gain

| Outcome | Prepregnancy BMI^*^ | | | |  | Rate of gestational weight gain^†^ | | |
| --- | --- | --- | --- | --- | --- | --- | --- | --- |
|  | Underweight | Normal weight | Overweight | Obese |  | Insufficient | Adequate | Excessive |
| Cesarean delivery | 0.88(0.79,0.99) | Reference | 1.69(1.47,1.95) | 2.14(1.53,2.98) |  | 0.94(0.83,1.06) | Reference | 1.12(1.01,1.24) |
| Preterm birth^‡^ | 1.16(0.96,1.39) | Reference | 1.01(0.80,1.29) | 0.92(0.54,1.57) |  | 1.36(1.10,1.68) | Reference | 1.32(1.10,1.59) |
| SGA | 1.79(1.46,2.18) | Reference | 0.79(0.58,1.08) | 0.76(0.40,1.44) |  | 1.47(1.17,1.83) | Reference | 0.72(0.58,0.89) |
| LGA | 0.60(0.49,0.74) | Reference | 1.93(1.58,2.35) | 3.42(2.33,5.02) |  | 0.78(0.62,0.98) | Reference | 1.54(1.30,1.83) |

Abbreviations: BMI, body mass index; CI: confidence interval; OR: odds ratio; SGA, small-for-gestational age; LGA, large-for-gestational age.

Values are odds ratios (95% confidence intervals). Adjusted for study centers, age, gestational age at delivery, education, drinking during pregnancy, passive smoking, annual household income, number of parity, gestational diabetes mellitus, pregnancy-induced hypertension, and gestational weight gain (continuous)^*^ or prepregnancy BMI (continuous) ^†^; Preterm birth was not adjusted for gestational age at delivery^‡^.
