# Supplementary material for: Association of prepregnancy body mass index, rate of gestational weight gain with pregnancy outcomes in Chinese urban women
Source: Nutr Metab (Lond). 2019 Aug 19;16:54. doi: 10.1186/s12986-019-0386-z (PMC6700840; doi:10.1186/s12986-019-0386-z)
Supplement: Supplementary file 5 — Table S5. Adjusted ORs (95% CIs) for pregnancy outcomes and rate of gestational weight gain. (DOCX 19 kb) [file 12986_2019_386_MOESM5_ESM.docx]

**Table S5.** Adjusted ORs (95% CIs) for pregnancy outcomes and rate of gestational weight gain

| Rate of gestational weight gain (kg/w) | Cesarean delivery | Preterm birth* | SGA | LGA |
| --- | --- | --- | --- | --- |
| All |  |  |  |  |
| P10: 0.25 | 0.88(0.80,0.95) | 1.22(1.06,1.41) | 1.52(1.29,1.79) | 0.80(0.69,0.92) |
| P25: 0.37 | 0.92(0.87,0.97) | 1.10(1.00,1.20) | 1.37(1.22,1.55) | 0.79(0.72,0.86) |
| P50: 0.50 | Reference | Reference | Reference | Reference |
| P75: 0.63 | 1.09(1.02,1.16) | 1.05(0.94,1.17) | 0.70(0.62,0.80) | 1.40(1.26,1.55) |
| P90: 0.78 | 1.17(1.07,1.27) | 1.42(1.24,1.63) | 0.57(0.47,0.70) | 1.82(1.59,2.07) |
| *P* _overall_ | <0.001 | <0.001 | <0.001 | <0.001 |
| *P* _nonlinear_ | 0.816 | <0.001 | 0.072 | 0.002 |
| Underweight |  |  |  |  |
| P10: 0.26 | 1.03(0.85,1.25) | 1.42(1.06,1.90) | 1.66(1.21,2.28) | 0.71(0.46,1.11) |
| P25: 0.38 | 0.94(0.83,1.06) | 1.12(0.92,1.35) | 1.63(1.30,2.04) | 0.67(0.53,0.85) |
| P50: 0.52 | Reference | Reference | Reference | Reference |
| P75: 0.67 | 1.13(0.98,1.31) | 1.15(0.90,1.47) | 0.54(0.42,0.71) | 1.80(1.32,2.47) |
| P90: 0.82 | 1.13(0.92,1.37) | 1.61(1.18,2.18) | 0.45(0.30,0.67) | 2.41(1.64,3.55) |
| *P* _overall_ | 0.409 | <0.001 | <0.001 | <0.001 |
| *P* _nonlinear_ | 0.250 | <0.001 | 0.034 | 0.133 |
| Normal weight |  |  |  |  |
| P10: 0.26 | 0.84(0.75,0.93) | 1.20(1.01,1.43) | 1.40(1.15,1.72) | 0.84(0.70,1.00) |
| P25: 0.37 | 0.93(0.87,0.99) | 1.10(0.98,1.23) | 1.21(1.03,1.40) | 0.81(0.73,0.90) |
| P50: 0.50 | Reference | Reference | Reference | Reference |
| P75: 0.63 | 1.07(0.98,1.15) | 1.07(0.93,1.22) | 0.82(0.69,0.97) | 1.40(1.23,1.59) |
| P90: 0.78 | 1.19(1.07,1.33) | 1.55(1.31,1.83) | 0.63(0.48,0.84) | 1.89(1.60,2.22) |
| *P* _overall_ | <0.001 | <0.001 | <0.001 | <0.001 |
| *P* _nonlinear_ | 0.687 | <0.001 | 0.951 | 0.008 |
| Overweight/Obese |  |  |  |  |
| P10: 0.20 | 0.91(0.72,1.14) | 0.97(0.67,1.40) | 1.12(0.68,1.84) | 0.84(0.63,1.11) |
| P25: 0.30 | 0.84(0.74,0.95) | 0.94(0.77,1.14) | 1.26(0.95.1.62) | 0.81(0.70,0.95) |
| P50: 0.41 | Reference | Reference | Reference | Reference |
| P75: 0.58 | 1.51(1.22,1.88) | 1.28(0.90,1.82) | 0.63(0.39,1.02) | 1.47(1.11,1.93) |
| P90: 0.72 | 1.72(1.32,2.24) | 1.59(1.06,2.40) | 0.62(0.36,1.10) | 1.49(1.07,2.07) |
| *P* _overall_ | <0.001 | 0.042 | 0.300 | 0.039 |
| *P* _nonlinear_ | 0.036 | 0.474 | 0.259 | 0.106 |

Abbreviations: BMI, body mass index; CI: confidence interval; LGA, large-for-gestational age; OR: odds ratio; SGA, small-for-gestational age.

Values are odds ratios (95% confidence intervals). Adjusted for study centers, age, gestational age at delivery, education, drinking during pregnancy, passive smoking, annual household income, and number of parity; Preterm birth was not adjusted for gestational age at delivery^*^.
