# Supplementary figures and images for: Association of prepregnancy body mass index, rate of gestational weight gain with pregnancy outcomes in Chinese urban women
Source: Nutr Metab (Lond). 2019 Aug 19;16:54. doi: 10.1186/s12986-019-0386-z (PMC6700840; doi:10.1186/s12986-019-0386-z)

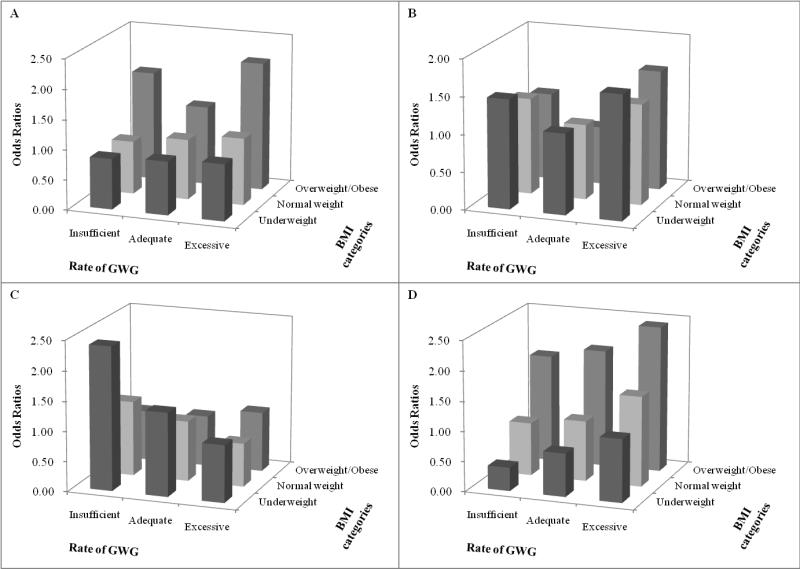

Supplement: Supplementary file 6 — Figure S1. Adjusted ORs for pregnancy outcomes according to prepregnancy BMI and rate of gestational weight gain. Abbreviations: BMI: body mass index; GWG, gestational weight gain; LGA, large-for-gestational age; OR: odds ratio; SGA, small-for-gestational age. Values are ORs for cesarean delivery (A), preterm birth (B), SGA (C) and LGA (D). Adjusted for study centers, age, gestational age at delivery, education, drinking during pregnancy, passive smoking, annual household income, and number of parity; Preterm birth was not adjusted for gestational age at delivery*. (JPG 52 kb) [file 12986_2019_386_MOESM6_ESM.jpg]
